# Converting Predation Cues into Conservation Tools: The Effect of Light on Mouse Foraging Behaviour

**DOI:** 10.1371/journal.pone.0145432

**Published:** 2016-01-13

**Authors:** Bridgette Farnworth, John Innes, Joseph R. Waas

**Affiliations:** 1 University of Waikato, Hamilton, New Zealand; 2 Landcare Research Manaaki Whenua, Hamilton, New Zealand; University of Sydney, AUSTRALIA

## Abstract

Prey face a conflict between acquiring energy and avoiding predators and use both direct and indirect cues to assess predation risk. Illumination, an indirect cue, influences nocturnal rodent foraging behaviour. New Zealand holds no native rodent species but has introduced mice *(Mus musculus)* that severely impair native biodiversity. We used Giving-Up Densities (GUDs) and observations of foraging frequency and duration to assess if artificial light induces risk avoidance behaviour in mice and could limit their activity. We found both captive (wild strain) mice in outdoor pens and wild mice within a pest fenced sanctuary (Maungatautari, New Zealand) displayed avoidance behaviour in response to illumination. In captivity, total foraging effort was similar across lit and unlit pens but mice displayed a strong preference for removing seeds from dark control areas (mean: 15.33 SD: +/-11.64 per 3.5 hours) over illuminated areas (2.00 +/-3.44). Wild mice also removed fewer seeds from illuminated areas (0.42 +/-1.00 per 12 hours) compared to controls (6.67 +/-9.20). Captive mice spent less than 1.0% of available time at illuminated areas, versus 11.3% at controls; visited the lit areas less than control areas (12.00 +/- 9.77 versus 29.00 +/-21.58 visits respectively); and spent less time per visit at illuminated versus control areas (8.17 +/-7.83 versus 44.83 +/-87.52 seconds per visit respectively). Illumination could provide protection at ecologically sensitive sites, damaged exclusion fences awaiting repair, fence terminus zones of peninsula sanctuaries and shipping docks that service offshore islands. We promote the hypothesis that the tendency of mice to avoid illumination could be a useful conservation tool, and advance knowledge of risk assessment and foraging under perceived danger.

## Introduction

Predation generates selective pressure for physiological, morphological and behavioural anti-predator adaptations [1, 2]. Foraging animals are at risk of predation while searching for, processing and consuming food and therefore employ a range of tactics to ensure survival, including predator avoidance behaviour [3]. However evading predators through avoidance can be energetically costly as it requires greater devotion to vigilance, reducing potential foraging time [4]. Prey consequently face a conflict between acquiring energy and avoiding predators [5]. The ability to behave flexibly and respond optimally to changes in predation risk is thus highly beneficial to prey as it prevents engaging in unnecessary vigilance [6]. Perceiving predation threats that fluctuate both temporally and spatially, and confining foraging activity to periods of low predation risk are components of the predation risk allocation hypothesis [7]. Animals perceive risk and manage activity to avoid predators by using both direct and indirect predation cues [8, 9].

Direct cues suggest the immediate presence of a predator [8] or act as an indicator of a specific type of predator [10] and include visual [11], tactile [12], auditory [13] and olfactory stimuli [14] associated with a predator. Reliance on direct cues may place the prey within close proximity of the predator and can therefore be dangerous, leading to the use of environmental and social factors (i.e. indirect cues) as surrogates for the level of risk posed [15]. Indirect cues inform prey of the probability of encountering a predator [8] and convey information regarding general predation risk [10]. Indirect cues include environmental factors such as microhabitat structure and ground colouration [10]. Social olfactory cues may also be indirect, such as alarm pheromones from disturbed, non-lethally injured or dead conspecifics [16].

One factor considered an indirect cue of predation risk is illumination [17], such as moonlight, which influences nocturnal rodent foraging behaviour [18–22]. Potentially, increased light intensity suppresses rodent activity because illumination enables better visual acuity by predators [23] and prey movements become more noticeable [24]. High movement under bright moonlight therefore magnifies prey susceptibility to predation [17]. Selection would favour individuals that decrease movement in light conditions favourable to predators [21].

Light avoidance in rodents has received limited attention from conservation biologists, with the exception of the influence of light pollution in deterring native rodent species from foraging areas [25]. Coastal beach lighting, for example, decreased the foraging behaviour of Santa Rosa beach mice *(Peromyscus polionotus leucocephalus)* in sand dunes [25]. New Zealand and other island ecosystems (e.g. Hawaii [26]) hold no native rodent species, but face a significant problem with non-commensal wild mice which can have serious consequences on native biodiversity [27–30]. Could artificial manipulations of light intensity provide conservation managers opportunities to control the activity of such pest species?

The house mouse (*Mus musculus*), is the smallest (mean weight 17–26 g) of the four rodent species introduced to New Zealand. Their diet includes invertebrates, fungal spores, lizards, birds and seeds [28]. Food supply generally limits mouse abundance, but mouse activity can be curtailed by other introduced predatory mammals such as rats (kiore *Rattus exulans*, Norway rat *R*. *norvegicus* and ship rat *R*. *rattus*), mustelids (stoat *Mustela erminea*, ferret *M*. *putorius*, and weasel *M*. *nivalis*) and cats (*Felis catus*) [31–34].

When released from predation and competition however, mice demonstrate capacity to inflict severe ecological damage. For example, mice are the single predatory mammal on Gough Island (South Atlantic) and strongly impact on seabird breeding success [35]. Despite their small size, wild mice preyed upon and killed large (ranging from 0.3 to 8kg), healthy seabird chicks (Atlantic Petrel *Pterodroma incerta*; Tristan Albatross *Diomedea dabbenena*), causing a steep decline in seabird populations [35, 36]. Angel [37] concluded that mice isolated from competition and predation could be as destructive as rats in their impact on avian wildlife.

Invasion by mice is a concern for predator-free islands and pest fenced sanctuaries where there is low predation pressure and low competition, as well as an abundance of food [27]. Mice are already the only mammal species present in several mainland sanctuaries protected by predator-proof fencing [38], either due to eradication survival [27] or their high propensity to successfully reinvade [39]. Predation by introduced mice causes significant changes to native species distributions, densities and persistence [37] generating concern for impacts that mice may have as the sole predatory mammals in New Zealand sanctuaries [27].

Although the food production industry has enlisted the use of deterrents for crop, fisheries and livestock protection with mixed results (see Bomford & O’Brien [40] for acoustic review; Koehler *et al*. [41] for visual and acoustic review; Apfelbach *et al*. [42] for olfactory review), few studies have sought to exploit predation cues in pest control or eradication for conservation purposes (though see Bramley *et al*. [43]; Shapira *et al*. [22]; Stober & Conner [44] for use of olfactory cues). Given the aforementioned properties of illumination on rodent behaviour [17], we assessed the utility of light as a deterrent for wild mice through inducing risk avoidance behaviour. Both captive trials and trials in a fenced sanctuary were used to assess how light influenced visitation rates at artificial foraging patches. Foraging strategies under illumination, such as whether mice increased visitation rates but decreased the amount of time per visit, were also examined. Fine-grained information about the small-scale movements of mice answer if light is a practical instrument for conservation or if mice would simply engage in alternative foraging tactics to fulfil their energy requirements. Both captive and field experiments not only advance the hypothesis that the behavioural tendency of mice to avoid illuminated areas could be useful as a conservation tool, but also expand current knowledge of risk assessment and engagement in optimal foraging under perceived danger.

## Methods

All methods were approved by the University of Waikato Animal Ethics Committee (Protocol 919). Approval for the study at Maungatautari was given by Maungatautari Ecological Island Heritage Committee. Permission was kindly granted for access across private land to the sampling site within the sanctuary by property owner Bill Garland and access to iwi land arranged by Tao Tauroa (Ngati Koroki Kahukura) and Robyn Nightingale (Raukawa Ki Wharepuhunga).

### Giving-Up Densities (GUDs)

Giving-Up Densities (GUDs) were initially advocated by Brown [45] for investigating the impact of factors such as predation risk, habitat preferences and interspecific competitive relationships on foraging behaviour. GUDs are useful for studying these relationships because they are based on optimal foraging theory yet incorporate real-time costs. These costs include the energy required to obtain resources (metabolic costs), the risk of detection by a predator (predation costs) and forgoing alternative food resources or mating opportunities to forage at a particular site (missed opportunity costs). An animal should therefore leave a patch with resources when the benefits of the harvest rate are less than the combined metabolic, predation and missed opportunity costs of foraging in the area [45]. GUD measurements are usually described by the number of food items remaining in a given foraging patch, however, due to the low rate of seed take in our study for clarity’s sake we simply report the number of seeds removed.

### Captive Experiments

#### Mouse curation

12 male adult C57BL/6 mice were sourced from Ruakura Research Centre (AgResearch), Hamilton, New Zealand. C57 is the wild type strain of laboratory mice, making them most appropriate for extrapolating to wild house mice. When not being used in tests, each mouse was housed indoors within a standard mouse cage (30 cm (L) x 20 cm (W) x 20 cm (H)), lined with 3 cm of wood shavings *(Pinus radiata)* and polyester nesting material. Subjects had access to standard mouse chow and water *ad libitum*; the light schedule was 12:12 light:dark at an intensity of 120 lux. Individuals used in tests were held in captivity for a period of ten weeks. All captive experiments were conducted in outdoor pens to have greater relevance to the subsequent field study. Therefore, prior to use in tests mice were housed at ambient outdoor temperature (between 10–15° c) for eight weeks to acclimatise. At this time mice were given trays with seeds and sand as used subsequently in trials, to condition mice to foraging in seed trays as well.

#### Experimental design

**Fig 1:**
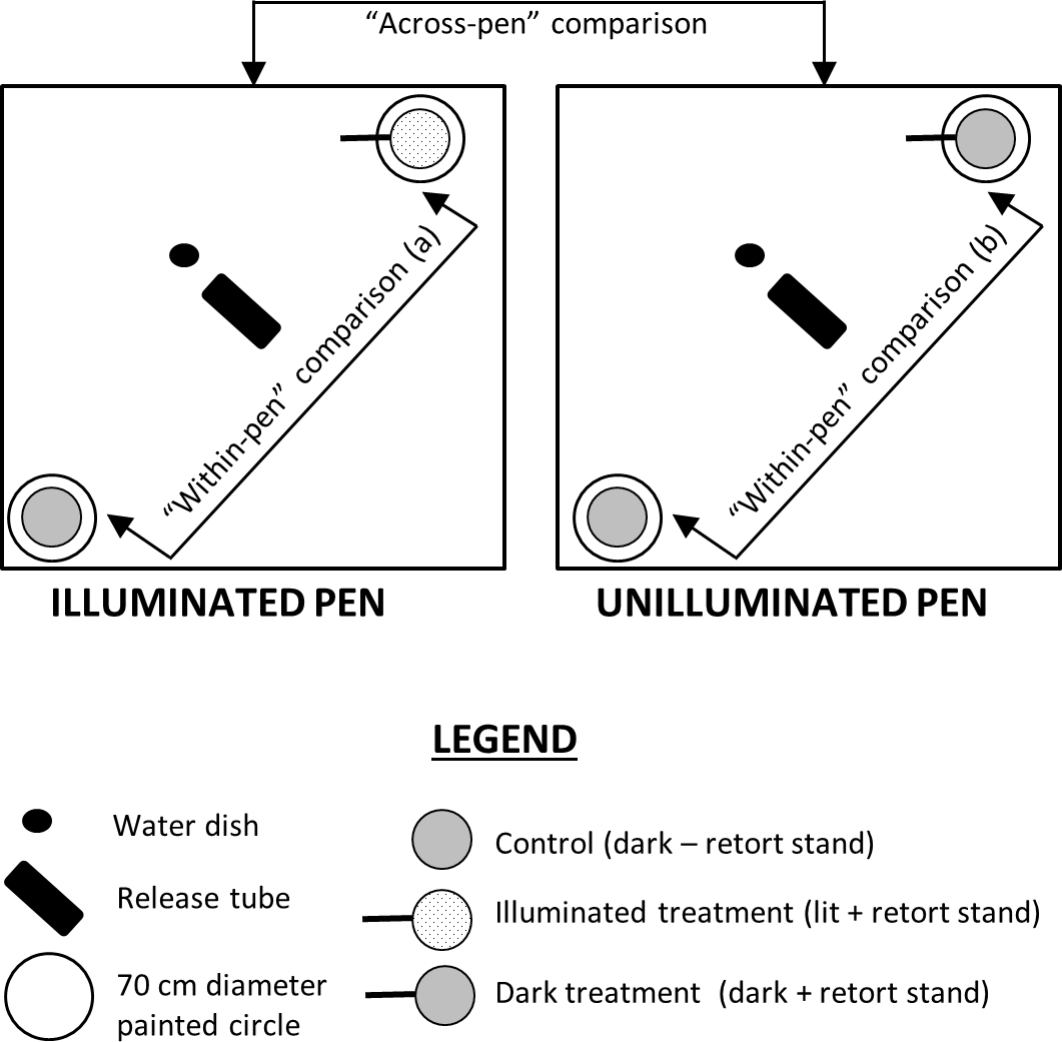
Bird’s eye view of the outdoor pens with three types of comparisons indicated (note: Illuminated treatment = seed tray in the centre of the painted circle underneath an illuminated light bulb that was suspended from a light stand; Dark treatment = seed tray in the centre of a painted circle underneath an unilluminated light bulb suspended from a light stand; Control = seed tray in the centre of a painted circle without illumination or associated equipment).

Tests in captivity took place within three outdoor pens at the University of Waikato, Hamilton, New Zealand. Each pen measured 4.0 m (L) x 3.2 m (W) x 2.0 m (H) and had a solid concrete floor; fine (6 mm^2^) wire mesh walls and ceiling rendered cages escape-proof. Black plastic weed mat covered walls and ceilings to prevent light incursion between pens and from buildings or the night sky. Within each pen, two circles (70 cm diameter) were marked with non-toxic, water-based paint two metres apart at opposite corners (Fig 1).

The circles enabled subsequent classification of when mice were ‘within’ or ‘outside’ the area of illumination and its unlit equivalent (Fig 1). To calculate the seeds removed, a small plastic ‘weigh tray’ measuring 11.5 cm (L) x 11.5 cm (W) x 2.5 cm (H) was used as a seed tray. Each seed tray contained 70 sunflower (*Helianthus* sp.) seeds with husks covered by 450 g of fine grain sand and was placed in the centre of each painted circle.

Each test was designed to present a subject with the choice of two seed trays that were two metres apart though the illumination conditions of each seed tray differed. For every pen, one seed tray was placed in a painted circle which was designated as the control. The control seed tray was in darkness and did not have any additional experimental equipment associated with it. The second seed tray was deemed the treatment seed tray and was placed in the opposing painted circle with one of two possible conditions (Fig 1):

Light:
A metal support stand with a downward facing illuminated light-emitting diode (LED) flood light (Ultra-LED, EQ3897/w, Electroquip, New Zealand; 12 volt; 3 watt) attached 60 cm above the ground. The circle of light produced was 1000 lux at ground level and 70 cm in diameter, reaching the edges of the painted circle on the ground. This condition is hereafter referred to as the illuminated treatment.
Dark:
A metal support stand with a downward facing *unilluminated* LED flood light attached, but otherwise identical to the above. This condition is hereafter referred to as the dark treatment. Note that the control seed tray was also dark but with no light support or floodlight.

The experimental design therefore allowed for two main types of comparisons to be made:

“Within-pen” comparison:
  - where the animal was simultaneously presented the choice of feeding from a lit seed tray (illuminated treatment) or a dark seed tray (control).
  - where the animal was simultaneously presented with the choice of feeding from a dark seed tray with unused lighting equipment (dark treatment) or another dark seed tray with none (control).

“Across-pen” comparison:
Where the feeding behaviour of an animal at the lit seed tray (illuminated treatment) on one test night was compared to the same animal’s behaviour at the dark seed tray (dark treatment) on a subsequent test night.

One individual painted circle was assigned to be a control; the other was systematically assigned to be either an illuminated treatment or a dark treatment, allowing determination of whether an individual favoured a particular corner. Within pens that contained one illuminated tray, the light intensity within each 70 cm circle was measured to ensure that illuminating one corner would not cause excessive light spillage into the opposing corner (illuminated circle: 1000 lux; control circle: < 2 lux). The individuals were assigned treatments in an alternating manner to compensate for order effects and individuals were never exposed to the same pen twice.

Each mouse was exposed to both the illuminated pen and the unilluminated pen, so that each subject completed two tests over an 8 day testing period. The individuals received three ‘rest’ days between tests to allow for recovery and to minimise any potential stress caused. Tests were 4 hours in duration and began within half an hour of sunset, as this is the usual foraging time for this species in the wild [46]. Six mice began in an illuminated pen while the remaining six began in an unilluminated pen. Each mouse was trialled in a different pen each night to reduce any influence which familiarity with an area may have on foraging behaviour. Foraging behaviour within each of the painted circles was recorded using video cameras (Color Weatherproof IR Camera, CCD 540 TVL, Lens 3.5–8 mm; Sony).

#### Test procedures

Three tests per evening were conducted from 13–20 September 2014 (austral spring). On the evening prior to exposure to a treatment, the mice which were to be released for the evening were individually placed into three larger cages (45.5 cm (L) x 32.0 cm (W) x 16.0 cm (H)) that each held a length of 30 cm PVC piping (referred to as the release tube). The larger cage also contained a seed tray with sand and 20 sunflower seeds to re-familiarize the mice to foraging within the seed tray. Captive individuals had continual access to standard mouse chow and water to replicate field trial conditions where food is plentiful for wild mice.

Within half an hour after sunset on the evening of each test, we removed the three release tubes containing the mice from the large holding cages and placed one tube at the marked position within each outdoor pen. Lights within pens assigned the illumination treatment were switched on prior to the release of the mice to remove any ‘startle’ effect on subjects. The release tube sat halfway between each of the treatment circles (Fig 1), with the exit of the tube perpendicular to the treatments so that mice were not directed towards a particular seed tray. Once the release tube was in place the subjects were able to freely exit and re-enter the tube and consume seeds *ad libitum* from either of the seed trays available within each pen for the duration of the test. While within the pens, the individuals had access to water at all times from a dish placed 15 cm from the exit of the release tube (Fig 1). At the conclusion of each test, we returned the subjects to their standard cages and sieved the contents of the seed trays to collect the remaining seeds. We counted and recorded intact seeds and washed the outdoor pens with 1:10 bleach to prevent odour traces. Seeds were considered to be removed by the subjects if they were wholly or partially consumed, or were displaced from the seed tray. Seeds were classified as ‘removed’ rather than ‘consumed’ because we could not determine if seeds were eaten rather than cached within the nesting material of the release tube.

#### Measures and data analysis

Four measures were recorded per trial: 1) the total time (seconds) an individual spent within each 70 cm circle; 2) the total number of times the individual entered each circle; 3) the average time (seconds) spent per visit; and 4) the number of seeds removed from each seed tray.

Both the number and duration of mouse visits to treatment and control circles were obtained by watching video playback. Due to failing recording equipment in some trials, the first half hour of each trial was eliminated from analysis to remove bias caused by missing data. This resulted in any statistical analysis using measures of time or frequency from 3.5 hours of footage for each test. A visit was deemed to have occurred when all four legs of a mouse were positioned within the circle. The total time (seconds) spent within the treatment area and control area was calculated per 3.5 hour time frame. The average amount of time (seconds) spent within each painted circle per visit was calculated by dividing the total duration of time by the number of visits.

For all measures, we assessed if data displayed a normal distribution (Shapiro-Wilks test) and homogeneous variance (Levene’s test). Dependent paired-t tests (or non-parametric equivalent: Wilcoxon Test) were conducted as appropriate in Statistica (version 12). “Within-pen” comparisons (Fig 1) analysed choices made by mice within a single enclosure and determined: (a) if mice displayed a preference for foraging in a dark versus illuminated tray; and (b) if the lighting equipment alone had an effect on the tray selected for foraging. “Across-pen” comparisons (Fig 1) analysed the influence that the presence of an illuminated versus unilluminated light system had on foraging behaviour within seed trays.

### Field Experiments

#### Study site

Mount Maungatautari (38°03’S, 175°33’W) is an eroded andesitic volcanic cone situated in the central Waikato area of the North Island of New Zealand [47]. The mountain supports a dense mix of podocarp-broadleaf species and forest types ranging from lowland forest dominated by rimu (*Dacrydium cupressinum*) and tawa (*Beilschmiedia tawa*), to montane forest composed of tawari (*Ixerba brexioides*), kamahi (*Weinmannia racemosa*), and tawheowheo (*Quintinnia serrata*) [47]. Maungatautari was selected as the study site in order to investigate the effect of illumination on mouse foraging in the absence of additional predators and competitors. Maungatautari provides an excellent field site for such trials as in August 2006 a 47-km Xcluder pest-proof exclusion fence (Xcluder^TM^ Pest Proof Fencing Ltd, Rotorua, New Zealand) encircling the 3363-ha area of Maungatautari was established [48]. Aerial poison application (‘Pestoff 20R’ cereal pellets, containing 20 ppm brodifacoum, Animal Control Products Ltd, Wanganui, New Zealand) followed by trapping and further poisoning was then used to kill all introduced mammals present [48]. Mice were initially also targeted for eradication, but since February 2012 no pest control targeting mice has been attempted and they have increased in abundance, remaining the only mammalian predator at the sanctuary [27]. Mouse abundance was monitored by index tracking (see Gillies & Williams, 2008) at Maungatautari. While mice initially had low abundance (10% tracking in December 2011), by December 2012 density has increased (approx. 70% tracking; Maungatautari Ecological Island Trust, Cambridge, unpub. data) and remained high through to at least August 2013 when monitoring ceased.

Research by Landcare Research Manaaki Whenua on Maungatautari suggests that the current 70% tracking using index tracking methods [49] indicates a mouse density of 10–20 per hectare [27], although mouse density may be patchy on the mountain.

#### Experimental design

We chose a study area on the western margin of Maungatautari that had pre-established marked routes suitable for setting up test locations and then established twelve experimental stations along four forest routes in homogeneous habitat that ranged from 340 m to 425 m in altitude.

Home ranges of wild house mice in New Zealand forests have been little studied though Fitzgerald *et al*. [29] give the average minimum home range area as 0.60 ha and the average range length as 123 m. The boundary of the average home range was at least 274 m long [29]. In sand dune habitat home ranges averaged 57.6 m in length [50]. Stations were therefore set up at 150 m intervals to minimise the chance of one home range overlapping two stations. Sites were placed approximately 5 m off the side of the track and at least 20 m from the nearest road to reduce edge effects.

Each forest track held three stations and each station was systematically assigned one of two treatments, though treatments were only applied to two of the tracks each night (Table 1). The two treatments used in field tests were:

**Table 1:** Treatment assignment to experimental stations at Maungatautari field sites where A(E) = acclimation night with experimental equipment only present at station; and A(E,S) = acclimation night with experimental equipment plus seed tray present at station; followed by two test nights where x = dark treatment; and o = illuminated treatment.

Forest Track	Station Number	Night 1	Night 2	Night 3	Night 4	Night 5	Night 6	Night 7	Night 8
	1	A(E)	A(E,S)	x	o				
**1**	2	A(E)	A(E,S)	o	x				
	3	A(E)	A(E,S)	x	o				
	1	A(E)	A(E,S)	o	x				
**2**	2	A(E)	A(E,S)	x	o				
	3	A(E)	A(E,S)	o	x				
	1					A(E)	A(E,S)	x	o
**3**	2					A(E)	A(E,S)	o	x
	3					A(E)	A(E,S)	x	o
	1					A(E)	A(E,S)	o	x
**4**	2					A(E)	A(E,S)	x	o
	3					A(E)	A(E,S)	o	x

Light:
A pigtail standard stand (90 cm standing height) with the same illuminated LED floodlight as used in the captive tests attached 60 cm above the ground. The circle of light produced was 1000 lux at ground level and 70 cm in diameter. This treatment is hereafter referred to as the illuminated treatment.

Dark:
A pigtail standard with an unilluminated flood light attached, but otherwise identical to the above and hereafter referred to as the dark treatment.

Depending on the slope of the terrain the height was altered in order to ensure the light intensity at ground level was 1000 lux (+/- 10 lux). The floodlight was contained within a custom made plastic cone that had water-proof sealing to prevent water damage to electrical fittings. The floodlight was fitted with three metres of cable and terminal clips to allow attachment to a 12 volt battery (Synergy; 40 amp hours). The battery was enclosed within a plastic bag to prevent water damage. At each station, the battery was placed three metres away from the upright pigtail standard in order to prevent: 1) casting a shadow over the illuminated area and 2) introducing bias due to animals avoiding a novel item placed close to a foraging patch.

Each station was used for four nights: two nights to acclimatise mice to equipment, and two trial nights with baited seed trays (Table 1). Initially the seed tray was presented with seeds after an initial equipment acclimatization night (Table 1). After one evening however no foraging had occurred at the seed trays, regardless of treatment. Therefore we added a lure to each seed tray for two final trial nights. Seed trays were baited using a small amount of peanut butter (Sanitarium; Australia) to cover a 10 cm² area on the inside of the bottom of the seed tray. 100 sunflower seeds were then added to the seed tray and covered with 200 g of fine grain sand. A small amount of peanut butter was then placed on a randomly chosen corner of the seed tray. This was to aid in attracting mice to the station and to assist in determining whether an animal had visited the station and taken the bait, but not persisted in foraging for seeds. On the first night (with lure), stations were assigned either dark or illuminated treatments, which were then reversed on the second night (Table 1).

#### Test procedures

The field study took place during 19–27 November, 2014 and corresponded to the period of a new moon to avoid interference by natural moonlight which is known to influence mouse foraging behaviour in New Zealand [22]. The weather was overcast during the testing period with overnight temperatures ranging between 8.5° c (min) and 15° c (max). Prior to the experiments, individuals received no previous habituation to consuming food items or to foraging within the seed tray. On the evening of the tests, stations were set with full seed trays at least 45 minutes before sunset and the floodlight was then connected to the battery terminals for illuminated treatments or left disconnected from the battery for dark treatments. After 12 hours (on the following day), we collected the seed trays in the same order in which they were set out, sieved the contents of the seed tray and counted the number of seeds which had been removed. Seeds were counted as removed if they were no longer within the plastic weigh tray or if they remained *in situ* but were no longer intact. Seeds were classed as ‘removed’ rather than ‘consumed’ because it could not be determined with certainty that seeds were eaten rather than cached at an unknown location.

#### Measures and data analysis

The number of seeds removed at stations which received the illuminated treatment was compared to the number of seeds removed at stations which received the dark treatment to determine if light influenced the foraging behaviour of mice. Data were analysed using non-parametric Wilcoxon tests, as the data were neither normally distributed (Shapiro-Wilks test) nor had homogeneous variance (Levene’s test). Statistical analysis was conducted using Statistica (version 12) software.

## Results

### Captive Experiments

#### “Within-pen” comparison (a): If given the choice within a single enclosure, do mice display a preference for foraging near a dark versus illuminated tray?

**Fig 2:**
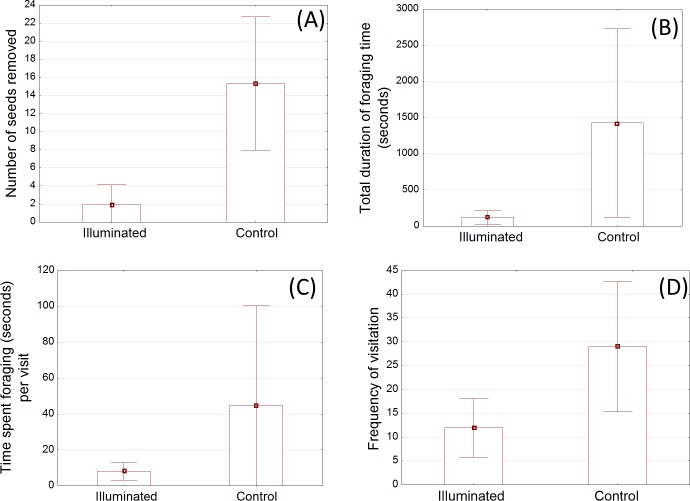
Within pen comparisons (mean with 95% confidence intervals) of the: (A) average number of seeds removed from the illuminated versus unlit seed tray; (B) total foraging time in an illuminated area compared with a dark control area; (C) mean amount of time spent foraging (seconds) per visit to near the illuminated seed tray compared with the dark control seed tray; and (D) frequency of visits by mice to the illuminated area compared with the dark control area over 3.5 hours.

When given a simultaneous choice between seeds in an illuminated seed tray and seeds in the unlit control tray, mice removed significantly more seeds from the control tray (p<0.01; Fig 2A).

Similarly, when presented with the choice between foraging near (defined as within the surrounding 70 cm circle) an illuminated tray versus an unlit control tray within a given pen, mice spent a significantly larger proportion of their total foraging time near the unlit control area (p<0.01; Fig 2B). On average, within a three and a half hour period, mice spent less than 1.0% of available time within the illuminated circle compared with 11.3% of their time within the control circle.

The amount of time spent per visit near the illuminated versus unlit control tray was also different (p<0.01; Fig 2C). Mice spent more than five times longer, per visit, near the unlit control tray than near the illuminated tray.

When presented with a choice between an illuminated tray and an unlit control tray within a given pen, mice visited near the unlit tray more than twice as often as the lit tray (p<0.05; Fig 2D).

Mice did not appear to change feeding effort across nights as there was no significant difference between the total number of seeds removed on the first test evening compared with the second test evening (p>0.1). The presence of light at one tray within a pen also did not diminish overall seed removal (p>0.1), as on average the total number of seeds removed within the light treated pens was very similar to the average removed within the totally dark pens (average total number of seeds removed from both seed trays within illuminated treatment pens = 17.33 +/- 12.96; average total number of seeds removed from both seed trays within dark treatment pens = 17.92 +/- 14.97).

#### “Within-pen” comparison (b): If given the choice within a single enclosure, are mice more likely to avoid a dark tray with experimental equipment adjacent to it than a dark tray alone?

No statistically significant difference was detected for the number of seeds removed (p>0.1), total duration of foraging time (p>0.1) or frequency of visitation (p>0.1) when mouse behaviour was compared at the seed trays in pens where neither of the trays was illuminated (i.e. control versus dark treatment). There was a difference between the amount of time spent per visit when the control was compared to the dark treatment though (p<0.05). Mice spent approximately two minutes on average per visit near the control tray but approximately half this time during visits to the dark treatment.

#### “Across-pen” comparisons: Does illumination reduce foraging behaviour near seed trays?

**Fig 3:**
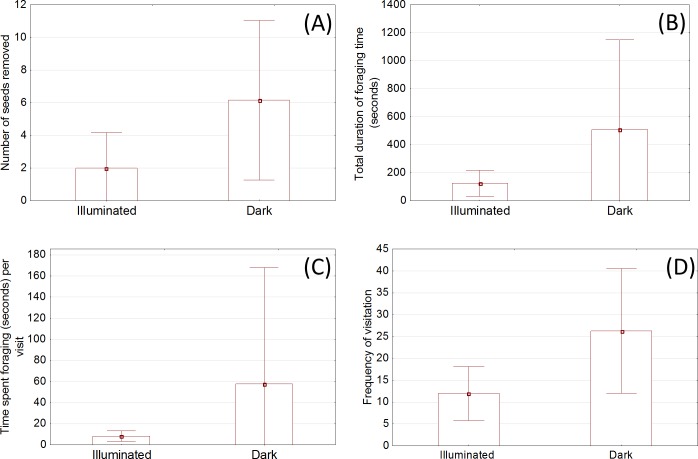
Across pen comparisons (mean with 95% confidence interval) of the: (A) average number of seeds removed from the illuminated area and dark treatment area; (B) total foraging time in the illuminated area and in the dark treated area; (C) time spent foraging per visit at the illuminated area and at the dark treated area; and (D) number of visits to the illuminated area and the dark treated area per 3.5 hour.

When comparing data across the two pens which mice were exposed to (i.e. pens where one tray was illuminated versus pens where neither tray was illuminated), the number of seeds removed was on average higher from the dark treatment seed tray compared with the illuminated seed tray, but the difference fell just short of being statistically significant (p = 0.066; Fig 3A).

Across pens, the total duration of foraging time was also greater, on average, in dark trays adjacent to the unlit bulb and stand than in lit trays. In pens with no illumination, mice spent more than three times as much time within the tray adjacent to the unlit bulb and stand than they did in the lit tray within illuminated pens, though the difference was not statistically significant (p>0.1; Fig 3B).

Across pen types, the amount of time spent foraging per visit was, on average, greatest near seed trays which were adjacent to unlit bulb. Mice spent seven times longer near unlit trays adjacent to the equipment than lit trays, but again, the difference was not statistically significant (p>0.1; Fig 3C).

Across pen types, the frequency of mice nearing an illuminated tray was three times lower than that for unlit trays adjacent to equipment, but the difference fell short of being statistically significant (p = 0.08; Fig 3D).

### Field Experiment

**Fig 4:**
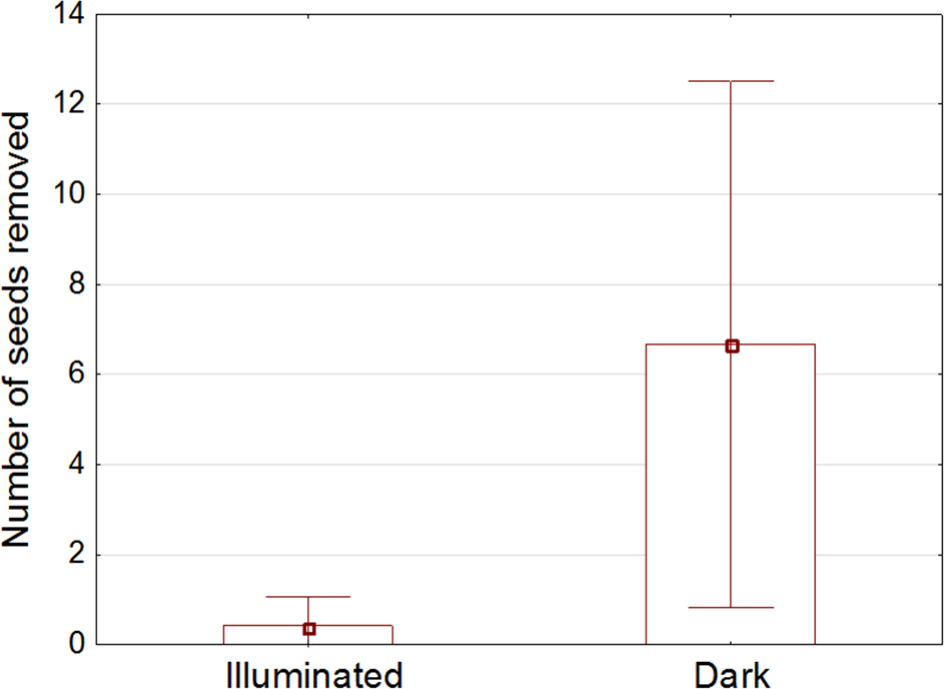
Comparison of the number of seeds removed from illuminated seed trays versus dark seed trays at field site per night (Maungatautari Ecological Island, New Zealand).

Fewer seeds were removed from illuminated than unlit stations (p<0.05; Fig 4) and mice did not appear to change feeding effort across treatment nights as there was no statistical difference in the total number of seeds removed on first versus second evenings (p>0.1).

## Discussion

We have experimentally demonstrated that both captive and non-commensal wild house mice exhibit avoidance behaviour in response to artificial illumination. Increases in natural light (e.g. moonlight) also reduce activity of wild house mice [9, 22]—mice in areas containing mammalian predators used sites with denser vegetation on moonlit compared to dark nights [9]. Also, a New Zealand study assessing the use of predator and competitor odours as a pest control strategy to deter mice, found that wild house mice foraged less on evenings with increased moonlight intensity [22]. The current research advances these studies as Dickman [9] and Shapira *et al*. [22] did not experimentally manipulate light levels and Shapira *et al*. [22] focused primarily on the impact of predator odour. Further, our captive experiments reveal how light influences foraging patterns. When presented with a simultaneous choice between an illuminated and a control seed tray captive mice: 1) display a preference for removing more seeds from the control tray; 2) spend more time overall foraging within the control area; 3) spend more time per visit in the control area; and 4) visit the control area more often.

Changes in the timing and frequency of foraging in response to altered light levels have been reported in wild prairie deer mice *(Peromyscus maniculatus bairdii;* Brillhart and Kaufman 1991) and two species of nocturnal Japanese field mice *(Apodemus speciosus*, *A*. *argenteus;* Sone 2002). In a captive environment all three species demonstrated behavioural strategies which, if used in natural habitat, would decrease the amount of time they were vulnerable to predators. Prairie deer mice reduced non-feeding activity under illumination, while Japanese field mice decreased the length and frequency of foraging excursions and concentrated foraging at sites close to safe refuges [19, 20]. In addition, resource use by both deer mice and Japanese field mice was similar in light and dark, as illumination did not decrease the total seed consumption in an evening [19, 20]. In our study, the total number of seeds removed per test (i.e. overall seed removal from both seed trays within a pen) by captive mice was also similar across lit and unlit pens. This corroboration between studies emphasises that illumination does not affect total resource consumption or removal, but instead affects where or when an individual obtains resources.

Individuals require adequate calories to maintain energy levels, but evidently are selective about the resource patches they exploit. If the perceived predation risk at one patch is too large, maximum fitness gains will occur by forgoing this cost and obtaining the same energy elsewhere. Thus, at Maungatautari, the unfavourable conditions caused by illumination resulted in foraging activity that shifted to locations which posed less risk. While the same pattern occurred in our captive trials, our carefully controlled experiment also revealed some avoidance of the unlit equipment (mice spent significantly more time per visit near the control than at the dark seed tray with experimental equipment); this suggests artificial structures near or overhanging foraging patches may also generate avoidance. Overall, however, our results show that foraging can be inhibited by artificial light, suggesting pest control applications for conservation biologists.

### Risk perception by nocturnal rodents

While we found mice reduced feeding activity in the presence of an indirect predation cue, wide variation existed in a subject’s willingness to enter the illuminated area. From a conservation perspective it is critical to know what factors influence an individual’s decision to forage, as any potential benefits of illumination could be counteracted by individuals that are willing to pursue activity in dangerous environments. Assessing the value of light as a management tool for reducing wild mice impacts therefore relies on further research considering risk perception by individual mice.

One factor shaping avoidance behaviour may be motivational status [51, 52]. Ecological theory attributes low motivation to pursue dangerous activities to satiation [51, 52]; individuals in good condition should be risk-averse and forage within safe habitats at low threat times, accepting lower quality food supply but remaining safe [53]. Conversely, individuals with poor body condition might avoid impending starvation by obtaining more profitable food items but increasing their probability of predation through foraging at riskier times or in higher-threat habitats [53]. This may drive under-nourished mice to persist in foraging for a greater reward despite the presence of predator cues indicating a high risk and cause pest control techniques using such cues to be ineffective against these individuals.

Previous encounters with predators and current predation pressure may also influence avoidance behaviour in response to illumination—rodents experiencing little or no predatory pressure can exhibit atypical responses to cues for increased predation risk [9]. For example, Dickman (1992) found mice inhabiting sites with avian predators but free of mammalian predators were less responsive to moonlight intensity than mice from sites where mammalian predators were present. However mammalian predators have been excluded from Maungatautari since 2006 when the sanctuary was fenced [48] and aerial hunters (e.g. morepork, *Ninox novaeseelandiae*) became the main predation threat. Field tests in the present study showed that mice at Maungatautari still avoided illuminated seed trays even in the absence of mammalian predators, contradictory to Dickman’s [9] results at Boullanger Island and Whitlock Island (Australia). Similarly Tawharanui Open Sanctuary (New Zealand) has excluded mammalian predators since 2004 [54], yet mice reduced foraging in seed trays during full moon periods with increased illumination [22]. Shapira *et al*. [22] theorised that the difference between responses observed at Tawharanui and at Boullanger and Whitlock Islands could have arisen because mice in Dickman’s [9] predator free sites had never been exposed to mammalian predators. Cats, red foxes *(Vulpes vulpes)* and western quolls *(Dasyurus geoffroii)* were never introduced to the island study sites used by Dickman [9] and were therefore historically absent in the context of influencing adaptive behaviour of prey species. Previous generations of mice at Tawharanui had exposure to mammalian predators though and this could have influenced anti-predator behaviour of their progeny [22]. However, the captive wild strain mice in our study had no exposure to predators, yet still displayed a preference for foraging in darkness compared with illumination. This suggests that the heritability of the response to illumination is high in mice and individual experience may be less important for shaping this particular anti-predator behaviour than suggested by Shapira *et al*. [22].

Mice occupying the Maungatautari sanctuary were from non-commensal populations, well established in natural habitats [29, 30]. While we showed that illumination does impact their foraging for at least short periods of time, the influence of illumination on truly commensal mice (cohabitating with humans and illumination) may produce different results; such individuals may continue foraging despite the presence of light and may be more likely to invade conservation areas near human settlements.

### Practical implications for conservation

The discovery that illumination provides point source deterrence against mice, even at highly profitable foraging patches, could have significant practical implications for conservation management. As demonstrated on Gough Island [36, 37], mice subjected to mesopredator release can be particularly damaging and similar impacts are speculated to occur in sanctuaries with exclusion fencing [27]. Illumination could therefore be particularly useful in protecting valuable nesting sites from mice. For example, fledgling veerys *(Catharus fuscescens)* were less likely to be predated on nights with a full-moon [55]. This short term improvement in avian survival [55] indicates a similar effect could be generated in small scale areas using artificial lighting. In addition, the combination of owl vocalisation and moonlight, had a significant effect on space use of white-footed mice, decreasing activity by nearly 67% [55]. The present study did not investigate the cumulative effects of predation cues, but suggests further research on combining auditory, olfactory, visual and tactile cues with illumination may result in beneficial conservation applications.

Illumination could also deter mice from breaches in exclusion fences until repair. While mainland exclusion fences have an excellent track record for keeping out other pest species, eradication and exclusion of mice frequently fails [38]. Connolly *et al*. [39] estimated that the potential for reinvasion by mammalian pests through pest-exclusion fencing in New Zealand was greatest 1) nocturnally, 2) from rodents and 3) in the summer time. In addition, illumination could protect peninsula sanctuaries by preventing reinvasion at fence terminus zones on shorelines. At Tawharanui Open Sanctuary for example, low tides expose up to 60 m of beaches allowing for potential pest incursions [54]. Mice also remain a threat to pest-free offshore islands as their removal would be a costly and time consuming operation [56]. Unwanted immigration to ecologically intact offshore islands may be minimised through the use of lighting at boat docks.

While managers should consider how to use aversive predation cues for conservation purposes, there may also be negative consequences. Research is required on whether mice would habituate to the light, as current studies on prey habituation to light are lacking. Commensal mice may rapidly habituate to illumination given their extensive persistence in human environments [30]. Our study was at a small scale; larger scale trials are needed. Illumination could also, as one study suggests, attract stoats due to their ability to find and capture prey more easily at illuminated sites [57]. Furthermore, illumination may interfere with valued native species. For example, light containing visible long-wavelength radiation attracted nocturnally migrating birds and disorientated them; lower visible long-wavelength sources of light however did not produce this effect [58]. There may therefore be combinations of both spectrum and intensity which are best to target pest species, while avoiding negative consequences for non-target species.
